# The Circadian Neuropeptide PDF Signals Preferentially through a Specific Adenylate Cyclase Isoform AC3 in M Pacemakers of *Drosophila*

**DOI:** 10.1371/journal.pbio.1001337

**Published:** 2012-06-05

**Authors:** Laura B. Duvall, Paul H. Taghert

**Affiliations:** Department of Anatomy & Neurobiology, Washington University Medical School, St. Louis, Missouri, United States of America; Baylor College of Medicine, United States of America

## Abstract

The neuropeptide Pigment Dispersing Factor (PDF) is essential for normal circadian function in *Drosophila*. It synchronizes the phases of M pacemakers, while in E pacemakers it decelerates their cycling and supports their amplitude. The PDF receptor (PDF-R) is present in both M and subsets of E cells. Activation of PDF-R stimulates cAMP increases in vitro and in M cells in vivo. The present study asks: What is the identity of downstream signaling components that are associated with PDF receptor in specific circadian pacemaker neurons? Using live imaging of intact fly brains and transgenic RNAi, we show that adenylate cyclase AC3 underlies PDF signaling in M cells. Genetic disruptions of AC3 specifically disrupt PDF responses: they do not affect other Gs-coupled GPCR signaling in M cells, they can be rescued, and they do not represent developmental alterations. Knockdown of the *Drosophila* AKAP-like scaffolding protein Nervy also reduces PDF responses. Flies with AC3 alterations show behavioral syndromes consistent with known roles of M pacemakers as mediated by PDF. Surprisingly, disruption of AC3 does not alter PDF responses in E cells—the PDF-R(+) LNd. Within M pacemakers, PDF-R couples preferentially to a single AC, but PDF-R association with a different AC(s) is needed to explain PDF signaling in the E pacemakers. Thus critical pathways of circadian synchronization are mediated by highly specific second messenger components. These findings support a hypothesis that PDF signaling components within target cells are sequestered into “circadian signalosomes,” whose compositions differ between E and M pacemaker cell types.

## Abstract 2

To synchronize a network of pacemakers in the *Drosophila* brain, a neuropeptide receptor specifically associates with adenylate cyclase 3 to create a “circadian signalosome.”

## Author Summary

In the fruit fly *Drosophila melanogaster*, the neuropeptide Pigment Dispersing Factor (PDF) supports circadian function by synchronizing two types of pacemaker cells, M cells and E cells. The PDF receptor (PDF-R) is a G protein coupled receptor (GPCR) whose activation stimulates adenylate cyclase (AC), thereby elevating levels of the second messenger cAMP in many different pacemakers including M cells. *Drosophila* contains at least 12 genes that encode potential ACs. In this study, we identify the AC downstream of the PDF receptor specifically in M cells and show that PDF signals preferentially through AC3. However, other GPCRs in the very same cells do not rely on AC3. A different scaffolding protein also influences PDF responses in M cells, suggesting that signaling components are spatially grouped to allow for coupling of specific receptors with downstream components. Remarkably, in E pacemakers, AC3 disruptions have no effect. These findings suggest that distinct PDF circadian signals exist in M versus in E pacemakers, and more generally, we propose a mechanism to differentiate signaling pathways that use common components.

## Introduction

The importance of biological rhythms in the anticipation and response to daily environmental changes is underscored by their conservation throughout nature. In eukaryotes, these rhythms are generated by a set of core clock genes that contrive to produce interlocked feedback loops. Both mammalian and *Drosophila* circadian rhythms are controlled by diverse groups of pacemaker neurons that express these core clock genes and proteins. In *Drosophila* these rhythms are required in ∼150 neurons, which can be subdivided into six bilateral anatomically distinct groups [1]. There appear to be two classes of pacemaker neuron in the fly brain that differ in many fundamental ways—these are termed M and E cells for historical reasons [2]–[4]. Previous work indicates that these subgroups are functionally as well as anatomically distinct and that certain cells are associated with specific components of daily locomotor behavior. Importantly, these associations are subject to specific environmental conditions, and they display considerable plasticity under different light and temperature conditions [2],[3],[5]–[8]. These pacemaker subgroups communicate to synchronize with each other to produce coherent circadian rhythms [9],[10]. Neuropeptides are critical mediators of intercellular communication between pacemaker cells in both mammals and *Drosophila* and a number are expressed in the *Drosophila* clock cell system, including the Pigment Dispersing Factor (PDF) [11]–[14].

Loss of the PDF peptide or its receptor leads to abnormalities in circadian locomotor behavior, including a reduction in morning anticipatory peak and a phase advance of the evening anticipatory peak under 12∶12 LD [14]–[17]. Under constant conditions these flies show high levels of arrhythmicity or short, weak rhythms. PDF controls the amplitude and phase of molecular rhythms of pacemaker cells [9],[18].

PDF's role in synchronization of clock cells indicates that its mechanism of action is largely within the cells of the clock network. The PDF neuropeptide is expressed by two specific pacemaker subgroups (large and small LNvs) and the PDF receptor is expressed widely, although not uniformly, throughout the circadian network in both M and E cell groups [19]. The PDF receptor signals through calcium and cAMP, although specific signaling components remain unknown [15],[17]. Signaling can be demonstrated in nearly all pacemaker cell groups in vivo [20]. Previous work indicates that M cells increase cAMP levels in response to at least two neuropeptides, PDF and DH31 [20]. The PDF and DH31 receptors belong to the same class II (secretin) G-protein coupled receptor (GPCR) family [21]. Both PDF [15],[17] and DH31 receptors [22] stimulate adenylate cyclases (AC) to produce cAMP in vitro, and in M cells in vivo [20], but the specific downstream components that differentiate the two peptide receptors remain unknown. Likewise, the basis for PDF's differential actions on the molecular oscillator in different pacemakers [9],[18] has not yet been explained.

The present study asks: What is the identity of downstream components that are associated with PDF-R signaling pathways in different circadian pacemaker neurons? Specifically, using live imaging of intact fly brains, we identify the particular adenylate cyclase (AC) isoform that is associated with PDF signaling in small LNv—commonly called M cells. Although some signaling components are common to both DH31 and PDF neuropeptide signaling, we report that DH31 signaling does not require the same AC in the small LNv cells. This suggests that PDF signals preferentially through its favored AC, while other GPCRs, in the same identified pacemaker neurons, couple to other ACs. In addition, AC3 manipulations have no effect on PDF-R expressing LNd cells, part of the E cell network. Thus in *Drosophila*, critical pathways of circadian synchronization are mediated by at least two, highly specific second messenger pathways.

## Results

**Figure 1:**
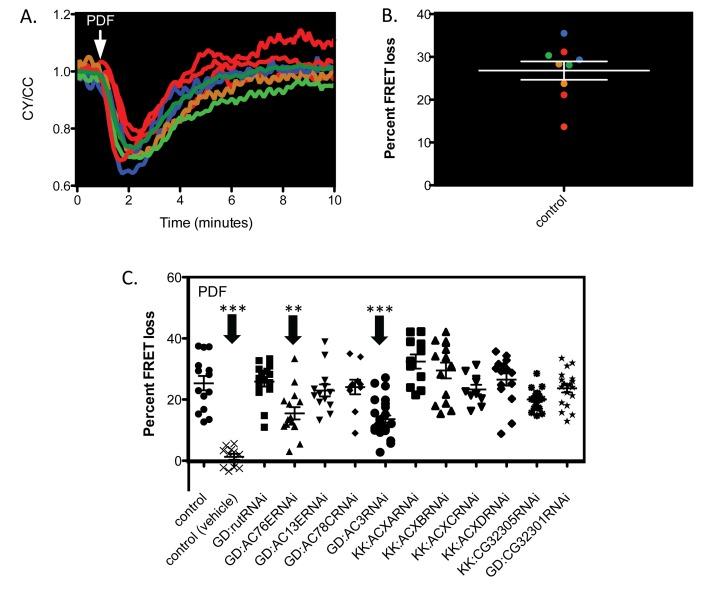
Data collection of FRET responses and transgenic RNAi screen of ACs potentially coupled to PDF receptor in M cell pacemakers. (A) Raw FRET imaging data (CY/CC) collected for 10 min (each trace represents an individual cell recorded as an ROI) to show FRET loss in response to a bolus of PDF (marked by arrow) and recoveries to baseline. (B) The scatter plot represents the data shown in 1A and retains its color-coding. For each trace, the maximal deflection from its value at the initial time point is computed as “percent FRET loss” and represented as a single point. Error bars represent SEM. (C) Double-stranded RNAi directed against 11/12 genes known to encode known adenylate cyclases in the *Drosophila* genome. All genotypes include *Pdf*-gal4;*Epac1camps* and one copy of UASRNAi (except for control). Error bars denote SEM. *** *p*<0.001, ** *p*<0.01 (compared with control).

*Epac1camps* is a genetically encoded cyclic nucleotide sensor that can be visualized with subcellular resolution and that responds with great sensitivity to cAMP in *Drosophila* neurons [20],[23]–[25]. Live brains expressing the reporter (using the gal4/UAS system) were imaged, while saline was perfused through the line and responses were measured to a bolus presentation of peptide (Figure 1A and B). M neurons were easily identifiable by their position and morphology using a *Pdf*-gal4 driver, and it was possible to obtain discrete readings from multiple cells within the same brain hemisphere.

In vitro assays indicate that *Epac1camps* has much higher sensitivity to cAMP than to other cyclic nucleotides [26]; however, it was also shown that the sensor responds to changes in cGMP levels at the *Drosophila* neuromuscular junction [23]. Based upon in vitro studies of PDF signaling, we hypothesized that PDF receptor activation leads to increases in cAMP, not cGMP, levels in these pacemakers [15],[17]. To test this idea, we used SNAP (S-Nitroso-N-acetyl-DL-penicillamine (1)) as a Nitric Oxide (NO) donor, which is known to stimulate cGMP production [27]. Addition of SNAP led to a measurable loss of the CFP/YFP FRET in M cells, consistent with the interpretation that the EPAC sensor detects increases in cGMP levels in addition to those of cAMP levels. SNAP responses were reduced in amplitude after pretreatment with a guanylate cyclase inhibitor 1H-[1],[2],[4]Oxadiazolo [4,3-a]quinoxalin-1-one (ODQ). Importantly, ODQ pretreatment had no effect on PDF responses. Genetic over-expression of a cAMP-specific phosphodiesterase *dunce* reduced the amplitude of PDF responses, but had no effect on SNAP responses in either M or E cells (Figures S1 and S2). Together, these results are consistent with the supposition that, in vivo, PDF signals through cAMP, not cGMP.

### Two Adenylate Cyclases (AC3 and AC76E) Score Positive in an In Vivo RNAi Screen Targeting Responses to PDF

The *Drosophila* genome encodes at least 12 ACs, five of which are expressed broadly, or at least broadly in the central nervous system (Flybase). The remaining cyclases (ACXA-E and CG32301 and CG32305) are thought to be expressed exclusively in the male germline ([28]; Flybase). *Rutabaga* (Rut) is the best characterized *Drosophila* ACs based on a mutagenesis screen for learning and memory phenotypes [29]. Rut is expressed in the *Drosophila* brain and is stimulated by calcium and calmodulin [30]. However, *rut* mutants showed normal PDF responses in both M and E cells (unpublished data), suggesting that a different AC(s) must mediate PDF-dependent signaling. Nevertheless, there is evidence that cAMP signaling is involved in circadian physiology in *Drosophila* [31]. Therefore, to test the role of other AC isoforms in PDF responses in small LNv cells, we performed a transgenic RNAi screen using constructs directed against 11 of the 12 ACs. Initial controls were performed with and without UAS-*dicer2*, however expression of *dicer2* alone showed nonspecific effects on PDF responses and therefore all experiments presented were performed without dicer expression (unpublished data). In M cells, two AC RNAi lines significantly reduced the amplitudes of PDF responses—*AC3* and *AC76E* (Figure 1C)—although in neither case were PDF responses completely abrogated (Figure 1C, compare to second column). In agreement with the initial *rut* mutant results, RNAi knockdown of *rut* mRNA had no effect on PDF responses. These results were consistent across different GAL4 lines (*Mai179*-gal4 and *tim*(UAS)-gal4) and therefore cannot be ascribed to differences in expression pattern or strength of the specific GAL4 driver used (unpublished data).

### AC3 Mediates PDF Signaling in M Cells in Adult Stages

**Figure 2:**
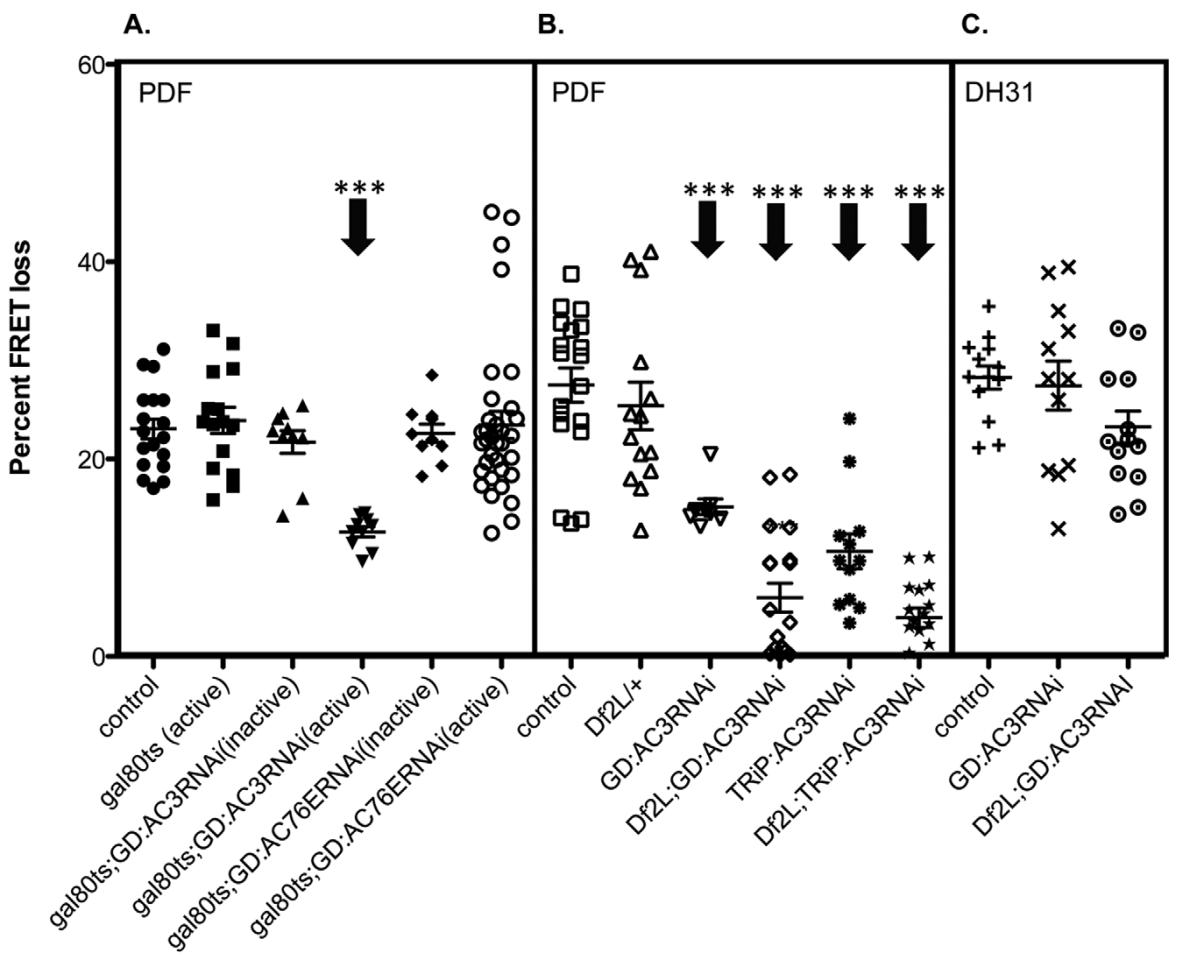
A conditional transgenic RNAi test of AC involvement in PDF signaling in M cell pacemakers. (A) Temperature sensitive gal80 was used to induce knockdown in adult cells only. Flies were raised at 18°C and moved to 29°C for 6 h (inactive), to allow for readable levels of *Epac1camps* sensor, or >36 h (active). Adult induction of *AC3*RNAi (gal80ts;*AC3*RNAi (active)) significantly reduces the PDF response. Adult induction of *AC76E*RNAi shows no significant difference from control. (B) Genetic confirmation of *AC3* involvement was performed using two independently generated RNAi lines against *AC3* (GD:*AC3* and TRiP:*AC3*) as well as flies that are deficient for the *AC3* gene region (*Df*(2L)*DS6*). (C) DH31 responses in M cells from flies with a knockdown of *AC3* in combination with *Df*(2L)*DS6*. All genotypes include *Pdf*-gal4;*Epac1camps*. Error bars denote SEM. *** *p*<0.001 (compared with control).

The results using *AC* RNAi could potentially be explained by deleterious effects on small LNv exerted by continuous RNAi expression throughout the neurons' period of development. To evaluate this possibility, we employed a temperature-sensitive genetic system that allows for normal development, followed by conditional induction of RNAi only in the adult fly. Animals raised at a permissive temperature (18°C) had gal4 activity blocked by a temperature-sensitive gal80 transgene (tubulin-gal80_ts_) [32]. After normal development, the flies were then moved to a higher temperature (29°C) at which the gal80 transgene is no longer active and the gal4 transgene can drive expression of the RNAi construct, as well as the *Epac1camps* sensor. When tested in this manner, adult-specific knockdown of *AC3*, but not of *AC76E*, resulted in a reduction of the PDF response in adult small LNv cells (Figure 2A). This indicates that developmental effects likely cause the reduction observed in the initial RNAi screen for *AC76C*, while the reduction observed for the case of *AC3* RNAi indicates its mediation of PDF responsiveness in adult small LNv pacemakers.

To further confirm AC3 as the candidate PDF-dependent AC and to exclude false positives (due to nonspecific RNAi knockdown), we performed further genetic tests using an independently generated *AC3* RNAi line from the Harvard TRiP project (TRiP:*AC3*RNAi) in addition to the line used in the initial screen from the VDRC (referred to as GD:*AC3*RNAi) [33] that targets a non-overlapping portion of the *AC3* RNA. The TRiP:*AC3* RNAi line also produced a significant decrease in the amplitude of PDF responses. In addition, both the VDRC and the TRiP:*AC3* RNAi lines were also tested in combination with flies that are deficient for the *AC3* gene region (*Df(2)LDS6*), to further reduce *AC3* levels. These *RNAi*/*Df* flies (hemizygous *AC3* mutants) showed a marked further reduction of the response to PDF neuropeptide in small LNv cells compared to responses in either single mutant genotype: *Df*/+ or *AC3* RNAi/+ (Figure 2B). Together these genetic experiments provide strong confirmation of our initial RNAi screening results and support the hypothesis that AC3 is the principal mediator of PDF-dependent signaling in small LNv cells. Importantly, the consequences of knocking down AC3 were highly specific to PDF: even when combined with the deficiency, AC3 RNAi had no effect on small LNv cell responses to a closely related cAMP-generating neuropeptide—DH31 (Figure 2C) [20]. This indicates that, in these same neurons, DH31-R likely signals through a different AC.

### Over-Expression of AC3, But Not Other ACs in M Cells Abrogates Their PDF Responses

**Figure 3:**
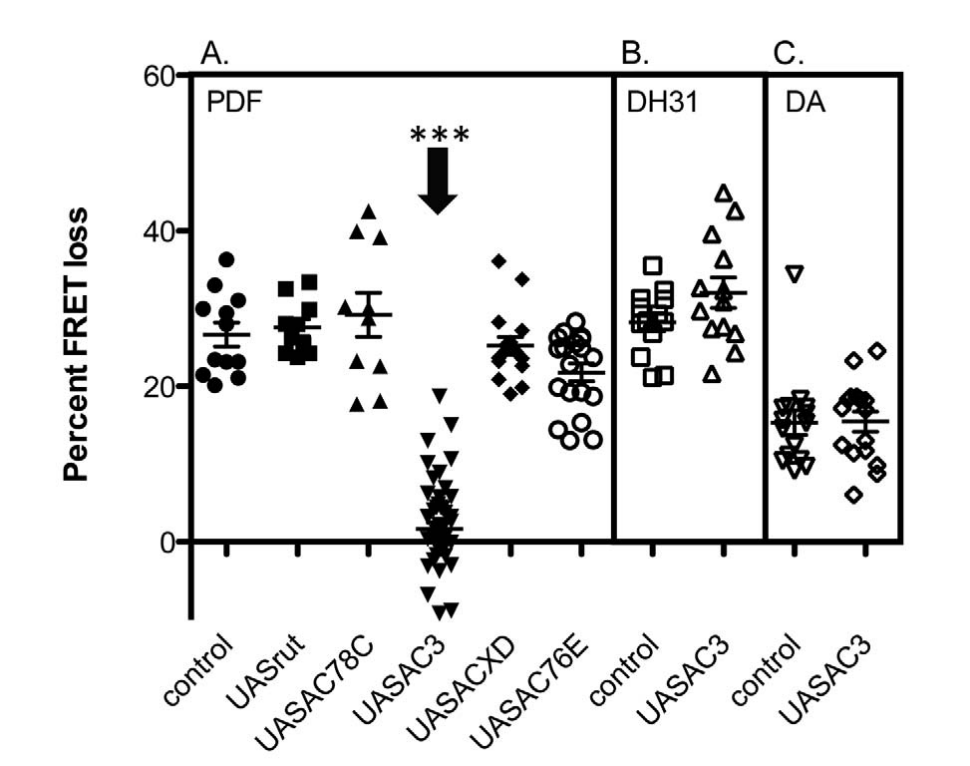
Effects of over-expressing *AC* isoforms on different receptor signaling systems in M pacemakers. (A) Effects of over-expressing diverse ACs on M cell responses to neuropeptide PDF. (B) Effects of over-expressing *AC3* on M cell responses to neuropeptide DH31. (C) Effects of over-expressing *AC3* on M cell responses to dopamine. All genotypes include *Pdf*-gal4;*Epac1camps*. Error bars denote SEM. *** *p*<0.001 (compared with control).

We tested the effects of UAS-*rut*, -*ACXD*, -*AC76E*, -*AC3*, and -*AC78C* to ask whether AC over-expression could affect PDF signaling in vivo. Novel constructs were first tested for functionality by measuring *cre*-LUC responses to 10 µM forskolin in *hEK* cells. All constructs tested showed an increased average response to forskolin compared to empty vector-transfected cells, although these did not reach significance (Figure S3). We were surprised to find that, in vivo, over-expression of *AC3* completely abrogated PDF responses in M cells, while over-expression of all other constructs had no such effect (Figure 3A). This disruption was not due to developmental effects: delaying UAS-*AC3* induction until the adult stage after completion of normal development (using the gal80ts system) produced the same disruption of PDF responses (Figure S4). In UAS-*AC3* flies, both DH31- and dopamine-elicited cAMP increases remained intact, indicating that the cells were demonstrably healthy and could respond normally to stimulation of other G*s*-coupled GPCRs (Figure 3B and C). These observations suggest that abnormally high levels of AC3 specifically disrupt the PDF signaling pathway and add further proof that AC3 is a unique component of PDF signaling in M cells.

### UAS-*AC3* Rescues the *AC3* RNAi Phenotype

**Figure 4:**
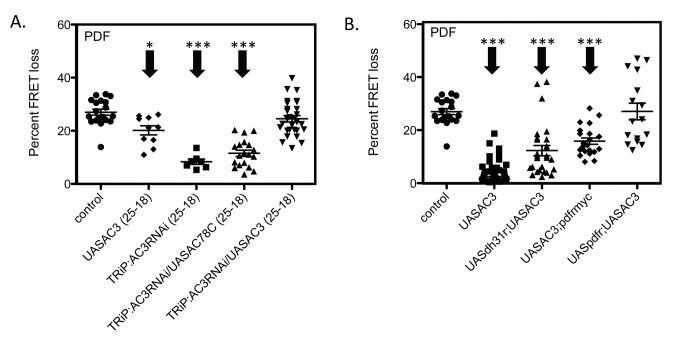
Genetic rescues of *AC3* knockdown and over-expression effects in M cells. (A) Rescuing the loss of function state. Flies were raised at 25°C and moved to 18°C as adults for 12–15 h before imaging to reduce levels of *AC3* over-expression. The effect of this schedule on the effects of *AC3* knockdown (TRiP:*AC3*RNAi) and *AC3* over-expression (UAS-*AC3*) is shown. The ability of over-expressing *AC78C* (TRiP:*AC3*/UAS*AC78C*) and *AC3* (TRiP:*AC3*RNAi/UAS*AC3*) to reverse the knockdown effect of *AC3* RNAi are also shown. (B) Rescuing the gain of function state. Two *PDF-R* over-expression genotypes were tested for their ability to affect *AC3* over-expression: a UAS construct (UAS*PDF-R*;UAS*AC3*) and a construct in which *PDF-R* is driven by its endogenous promotor (UAS*AC3*;*PDF-R*myc). For comparison the effects of co-misexpressing a heterologous neuropeptide receptor is also shown (UAS*DH31R*/UAS*AC3*). All genotypes include *Pdf*-gal4;*Epac1camps*. Error bars denote SEM. *** *p*<0.001, * *p*<.05 (compared with control).

Knocking down *AC3* levels produced a diminution of PDF signaling in M cells (Figure 2): to evaluate further the specificity of this effect we wished to employ an *AC3* rescue strategy. However, over-expressing the AC3 enzyme in M cells above normal levels disrupted M cell responsiveness to PDF (Figure 3A), suggesting that supra-normal levels of the AC3 enzyme can also lead to dysfunction. Therefore, we reasoned that a successful design to rescue the *AC3* knockdown would require a more moderate level of *AC3* over-expression. Because the gal4 system is temperature-sensitive, intermediate levels of *AC3* over-expression were achieved by raising the flies at 25°C and then moving them to 18°C overnight before imaging. This temperature shift could reduce the activity of the gal4 driver, which could result in lower levels of UAS-*AC3* expression. Indeed this schedule of temperature changes reduced the disruptive effect of AC3 over-expression on responses to PDF in M cells (Figure 4A, second column). We wondered whether it could also maintain effective RNAi knockdown of the endogenous gene.

We confirmed that firstly the RNAi transgene is still active under this temperature regimen (Figure 4A, third column). This UAS-*AC3* RNAi line is directed against the 3′ untranslated region (UTR) of *AC3* and can therefore be rescued potentially by expression of UAS-*AC3*, which includes only the *AC3* coding region. In fact, over-expression of UAS-*AC3*, with a temperature shift from 25°C to 18°C at adulthood, rescued the reduction in PDF responses otherwise observed in a UAS-*AC3* RNAi line (Figure 4A). Comparable over-expression of *AC78C* did not rescue this deficit and that result also confirms that the rescue was not due to simple dilution of the gal4 driver. Importantly, temperature down-shifted (25°C to 18°C) over-expression of *AC3* alone, which should result in a small overshoot of normal enzyme levels, shows a slight reduction in PDF responses compared to control (Figure 4A). This again suggested that normal levels of receptor and enzyme are key for normal function. Together these results provide strong evidence to support the hypothesis that AC3 is a specific AC isoform in M cells whose levels are tightly controlled and that normally mediates responsiveness to PDF signaling.

### Over-Expressing PDF Receptor Rescues Loss of Responsiveness to PDF due to Over-Expressed AC3

We pursued the AC3 over-expression condition to further evaluate the nature of the components of the PDF receptor signalosome in M cell pacemakers. We reasoned that we could perhaps counteract an imbalance between signaling components produced by AC3 over-expression if we also over-expressed the PDF receptor. In fact, over-expressing PDF-R using a UAS-*PDF-R* transgene in combination with UAS-*AC3* fully rescued the PDF response back to control levels (Figure 4B). The combination of *AC3* over-expression with an additional copy of *PDF-R* (under control of its own promoter within a ∼70 kB transgene, termed *PDF-R*-*myc*; [19]) produced a partial rescue of the PDF response. The latter effect was smaller than that seen with UAS-*PDF-R*, presumably because the induced level of *PDF-R* over-expression was greater with the UAS construct. Co-misexpression of the closely related neuropeptide receptor *dh31-R1* (CG17415; [22]) along with *AC3* also gave a partial rescue of diminished PDF signaling due to AC3 over-expression, although these responses were still significantly lower than control and less than what we observed with co-misexpression of *PDF-R* and *AC3*. Together, these results suggest that (i) the diminution of PDF signaling that follows AC3 over-expression can be rescued by providing more PDF receptor, thus reducing the receptor/effector imbalance. It also suggests that (ii) the absolute ratio of PDF receptor to AC3 enzyme is important for normal neuropeptide signaling in M cells.

### *AC3* Knockdown Does Not Affect All Gs-Coupled GPCR Signaling in M Cells

**Figure 5:**
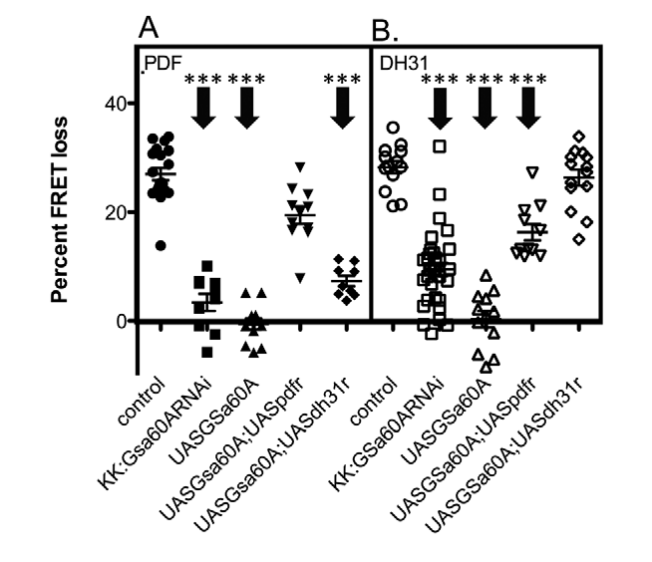
Both PDF and DH31 responses are affected by altering G_s_α60A levels. (A) PDF responses following knockdown or over-expression of G_s_α60A. G_s_α60A over-expression effects were also measured in the context of over-expression of PDF-R (UAS*G_s_* ***α*** *60A*;UAS*PDF-R*) or over-expression of DH31R (UAS*G_s_* ***α*** *60A*;UAS*DH31R*). (B) DH31 responses following knockdown or over-expression of *G_s_α60A*. *G_s_α60A* over-expression effects were also measured in the context of over-expression of PDF-R (UAS*G_s_* ***α*** *60A*;UAS*PDF-R*) or over-expression of DH31R (UAS*G_s_* ***α*** *60A*;UAS*DH31R*). All genotypes include *Pdf*-gal4;*Epac1camps*. Error bars denote SEM. *** *p*<0.001, * *p*<.05 (compared with control).

Both RNAi and over-expression screens suggested that PDF receptor associates preferentially to the AC3 adenylate cyclase in M cells, although expression profiling studies indicate that multiple AC isoforms are expressed in these identified pacemakers [34]. To determine the specificity of AC3 contributions to other peptide signaling pathways in M cells, we evaluated cAMP responses produced by other ligands for Gsα coupled GPCRs. *Drosophila* DH31 (Diuretic Hormone 31) is a neuropeptide closely related to mammalian Calcitonin, and the DH31 receptor (CG17415) is closely related to the Calcitonin receptor [22]. Activation of PDF receptor and DH31 receptor both lead to increases in cAMP and hence both are presumably coupled to Gsα [17],[22]; both increase cAMP in M cells in vivo [20]. RNAi knockdown of the Gsα60A subunit disrupted both signaling pathways, as expected. Interestingly, over-expression of the *Drosophila* G protein Gsα60A also disrupted both PDF and DH31 signaling in M cells, and responses could be restored by over-expression of the cognate receptor along with Gsα60A (Figure 5A and B). As mentioned above, neither knockdown nor over-expression of *AC3* affected DH31 responses (Figure 2C). We interpret these results to suggest that both PDF and DH31 receptors are coupled to Gsα60A, but that PDF-R subsequently signals through AC3 and DH31-R through a different AC.

### Knockdown of AKAP *Nervy* Reduces PDF Responses

**Figure 6:**
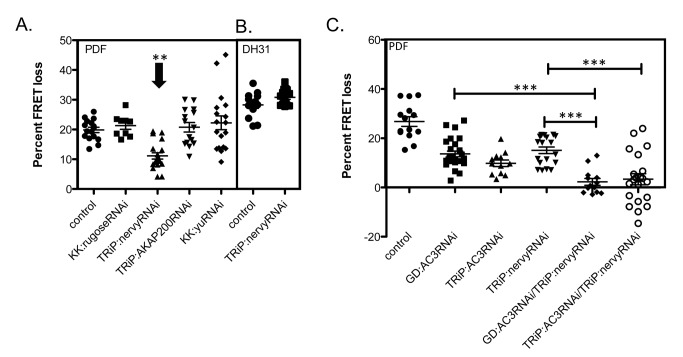
Effects on PDF responses following RNAi knockdown of scaffolding protein RNAs in M cells. (A) PDF responses of M pacemakers in flies expressing *nervy* RNAi. (B) DH31 responses of M pacemakers expressing *nervy* RNAi. (C) PDF responses of M pacemakers expressing *AC3* and *nervy* RNAi. All transgenic lines are significantly different (<.001) from control and internal comparisons are highlighted by bracketed lines. All genotypes include *Pdf*-gal4;*Epac1camps*. Error bars denote SEM. *** *p*<0.001 (compared with control).

Scaffolding proteins play important roles in supporting assembly of specific signalosomes, which feature tight association between specific receptors and specific second messenger molecules [35]. We hypothesized that scaffolding proteins may help explain the preference of PDF-R for coupling to AC3. In *Drosophila* there are four known AKAP (A-kinase anchoring proteins), molecules that bind to and help co-localize many components of cAMP signaling pathways [35]. We tested the possible involvement of AKAPs as scaffolding proteins for PDF-R in M cells using gene-specific RNAi constructs. Knockdown of the AKAP *nervy*, but not of the other three AKAPs, reduced PDF responses to an extent similar to that produced with the AC3 RNAi (Figure 6A). As with *AC3*, *nervy* knockdown showed no effect on DH31 responses in M cells (Figure 6B). When both *AC3* and *nervy* are knocked down together in the same M cell, PDF responses were disrupted to an even greater extent than with either RNAi alone (Figure 6C). The results from single versus double RNAi constructs were generally consistent, although the comparison between TRiP:*AC3*RNAi and TRiP:*AC3*RNAi/nervyRNAi does not reach significance (Figure 6C). This suggests that nervy also plays a role in PDF signaling in small M cells, presumably by allowing PDF signaling components to effectively localize and thus promote efficient signaling.

### *Gsα* Alterations Affect E Cell PDF Responses But *AC3* Alterations Do Not

**Figure 7:**
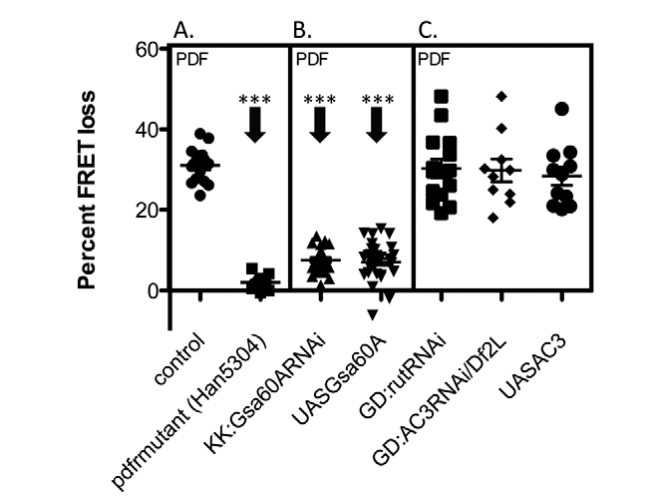
Effects of manipulating *G_s_α60A* and AC3 levels in E cell subgroup. (A) PDF responses in PDF-R expressing LNd cells (E cells). Flies with the severe PDF-R mutation *han^5304^* show no response to PDF. (B) PDF responses in PDF-R expressing LNd cells (E cells). Both knockdown and over-expression of *G_s_α60A* significantly reduce PDF responses in E cells. (C) PDF responses in PDF-R expressing LNd cells (E cells) in genotypes that most severely disrupt M cell PDF responses. Knockdown (*Df*(2L)/*AC3*RNAi) and over-expression of AC3 do not affect E cell PDF responses. All genotypes include *Mai179*-gal4;*Epac1camps*. Error bars denote SEM. *** *p*<0.001 (compared with control).

A number of previous studies have suggested that PDF signaling pathways differ between M and E cells. We therefore investigated PDF-R expressing LNd cell (the CRY+/PDF-R+ subset of LNd, using the Mai179-gal4 driver [18],[19],[36]) to evaluate the role of AC3 in E cell PDF signaling. We first confirmed that PDF-induced cAMP responses in these neurons are dependent upon PDF-R; flies with the strong *PDF-R* mutation (*han^5304^*) totally lose E cell responsiveness (as has been previously reported in M cells) [20]. As in M cells, Gsα manipulations again reduced PDF responses in E cells (Figure 7B). Previous experiments confirmed that ACs are involved in E cell PDF responses (Figure S2). The first AC we tested was *rutabaga*, which had proven ineffective in reducing PDF responses in M cells (Figure 1C). E cells in *rut* mutants produced normal PDF responses (unpublished data); responses to PDF were likewise normal following *rut* RNAi expression (Figure 7C). In the case of *AC3*, neither RNAi knockdown (combined with a *AC3 Df*) nor *AC3* over-expression altered PDF responses in this E-type clock cell subgroup. E cell responses were robust even though these same genetic manipulations produced the most severe reductions in M cell PDF responses (compare Figure 7C with Figure 2B and Figure 3A). Notably, M cell responses were reduced even when measured in the same brains in which E cells proved responsive (unpublished data).

### AC3 Alterations Affect Circadian Behavior

**Figure 8:**
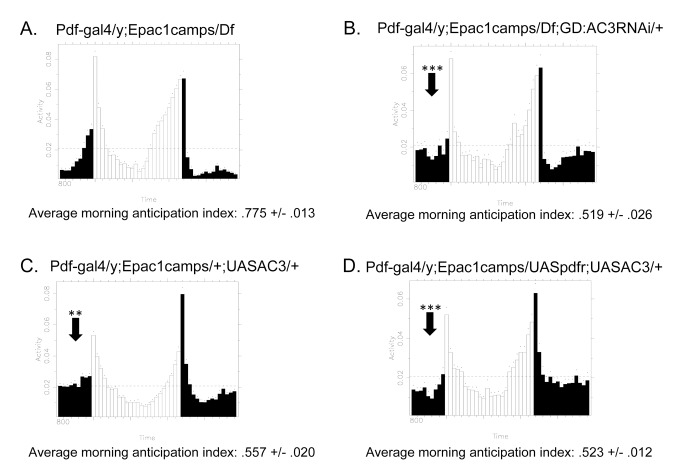
Effects on circadian locomotor activity of altering AC3 in M pacemakers. (A) Representative locomotor behavior of flies that are heterozygous for the *AC3* locus (*Df*(2L)*DS6*). (B) Representative locomotor behavior of flies that combine a knockdown of *AC3* by RNAi together with a deficiency for the *AC3* locus. (C) Representative locomotor behavior of flies over-expressing *AC3*. (D) Representative locomotor behavior of flies over-expressing *PDF-R* and over-expressing *AC3*. Morning anticipation index was calculated as (sum of activity 3 h before lights-on)/(sum of activity 6 h before lights-on). The average morning anticipation index was calculated from three replicates for each genotype. Error bars denote SEM. *** *p*<0.001 (compared with control). Statistical analysis of morning anticipation is shown in Table 1, and behavioral outcomes for DD are shown in Table 2.

The foregoing data argue that AC3 mediates the cAMP generation produced by PDF in M cells. To what extent is circadian locomotor behavior affected by this disruption of this AC3 activity? Manipulations that partially reduced M cell responses to PDF (e.g., RNAi knockdown of any single AC or AKAP) did not affect locomotor rhythms (see Figure S5 and Tables 1 and 2). However, combining *AC3* RNAi knockdown with a deficiency for the *AC3* region produced a very strong reduction in the morning anticipation peak, as well as higher levels of arrhythmicity under constant conditions (Figure 8B and Tables 1 and 2). We observed the same effects in UAS-*AC3* over-expression in PDF cells (Figure 8C and Tables 1 and 2). Over-expression of UAS-*PDF-R and UAS-AC3* together slightly reduced arrythmicity in DD compared to UAS-*AC3* alone (Table 2). However, the loss of morning anticipation seen in the UAS-*AC3* condition is not rescued by over-expression of the PDF receptor (Figure 8D and Table 1). This suggests that, although the PDF FRET response is rescued (Figure 4B), additional (for example, temporal) aspects of PDF signaling may contribute to normal circadian behavior in LD.

**Table 1:** Quantification of Morning Anticipation Index for LD behavior.

Tukey's Multiple Comparison Test	Mean Diff.	q	*p*<0.05?	Summary	95% CI of Diff
control versus *Pdf^01^*	0.312	9.171	Yes	***	0.1416 to 0.4824
control versus *Df*(2L)/+	0.014	0.4115	No	*ns*	−0.1564 to 0.1844
control versus *Df*(2L)/GD:*AC3*RNAi	0.2703	7.947	Yes	***	0.09997 to 0.4407
control versus UAS*AC3*	0.2317	6.81	Yes	**	0.06130 to 0.4020
control versus UAS*PDF-R*;UAS*AC3*	0.2663	7.829	Yes	***	0.09597 to 0.4367
control versus GD:*AC3*RNAi	0.079	2.322	No	*ns*	−0.09137 to 0.2494
control versus TRiP:*AC3*RNAi	0.1893	5.566	Yes	*	0.01897 to 0.3597
control versus GD:*AC76E*RNAi	0.041	1.205	No	*ns*	−0.1294 to 0.2114
control versus TriP:*nervy*RNAi	0.2237	6.575	Yes	**	0.05330 to 0.3940
*Pdf^01^* versus *Df*2L/+	−0.298	8.76	Yes	***	−0.4684 to −0.1276
*Pdf^01^* versus *Df*2L/GD:*AC3*RNAi	−0.04167	1.225	No	*ns*	−0.2120 to 0.1287
*Pdf^01^* versus UAS*AC3*	−0.08033	2.361	No	*ns*	−0.2507 to 0.09003
*Pdf^01^* versus UAS*PDF-R*;UAS*AC3*	−0.04567	1.342	No	*ns*	−0.2160 to 0.1247
*Pdf^01^* versus GD:*AC3*RNAi	−0.233	6.849	Yes	**	−0.4034 to −0.06263
*Pdf^01^* versus TRiP:*AC3*RNAi	−0.1227	3.606	No	*ns*	−0.2930 to 0.04770
*Pdf^01^* versus GD:*AC76E*RNAi	−0.271	7.966	Yes	***	−0.4414 to −0.1006
*Pdf^01^* versus TRiP:*nervy*RNAi	−0.08833	2.597	No	*ns*	−0.2587 to 0.08203

Statistical analysis of morning anticipation behavior calculated as (total activity 3 h before lights-on)/(total activity 6 h before lights-on) for genotypes shown in Figure 8 and Figure S5. Average morning anticipation was calculated from three replicates. *Pdf^01^* genotype represents *Pdf*-null mutants, which have been widely studied and serve as an example of a total lack of morning anticipation.

*: *p*<0.05,

**: *p*<0.01,

***: *p*<0.001. *ns*, not significant.

**Table 2:** DD behavioral outcomes grouped by genotype.

Genotype	*n*	% Arrhythmic	Period (h) ± SEM	Power ± SEM
*Pdf*-gal4/y;*Epac1camps*	59	2	24.3±0.09	81.7±3.6
*Pdf-*gal4/y;*Epac1camps*/*Df*(2L)	28	11	23.7±0.08	64.7±8.7
*Pdf*-gal/y;*Epac1camps*/*Df*(2L);GD:*AC3*RNAi/+	33	82	23.9±0.20	39.7±8.6
*Pdf*-gal4/y;*Epac1camps*/+;UAS*AC3*/+	46	83	24.0±0.45	26.9±5.2
*Pdf* -gal/y;*Epac1camps*/UAS*PDF-R*;UAS*AC3*/+	39	64	24.3±0.40	23.2±4.0
GD:*AC3*RNAi/+	29	7	23.6±0.12	70.2±5.5
TRiP:*AC3*RNAi/+	39	20	23.3±0.05	43.9±4.6
*Pdf*-gal4/y;*Epac1camps*/+;GD:*AC3*RNAi/+	31	0	24.4±0.07	84.0±4.1
*Pdf*-gal4/y;*Epac1camps*/+;TRiP:*AC3*RNAi/+	30	3	24.1±0.07	81.6±6.7
*Pdf*-gal4/y;*Epac1camps*/GD:*AC78C*RNAi	54	6	24.1±0.06	88.7±4.1
*Pdf*-gal4/y;*Epac1camps*/+;GD:*rut*RNAi/+	40	10	24.3±0.07	87.8±4.5
*Pdf*-gal4/y;*Epac1camps*/GD:*AC13E*RNAi	25	4	24.2±0.14	85.4±6.6
*Pdf*-gal4/y;*Epac1camps*/+;GD:*AC76E*RNAi/+	52	2	24.2±0.06	99.0±3.9
*Pdf*-gal4/y;*Epac1camps*/KK:*ACXA*RNAi	17	0	24.5±0.16	46.0±4.1
*Pdf*-gal4/y;*Epac1camps*/KK:*ACXB*RNAi	29	14	24.7±0.23	101.6±5.8
*Pdf*-gal4/y;*Epac1camps*/KK:*ACXC*RNAi	22	17	23.7±0.09	75.8±14.7
*Pdf*-gal4/y;*Epac1camps*/KK:*ACXD*RNAi	20	0	24.3±0.13	111.5±8.7
UAS*AC3*/+	24	8	23.8±0.18	56.2±9.1
*Pdf*-gal4/y;*Epac1camps*/UAS*AC76E*	30	20	24.4±0.11	59.4±5.6
*Pdf*-gal4/y;*Epac1camps*/+;UAS*AC78C*/+	69	16	24.6±0.07	57.0±3.9
*Pdf*-gal4/y;*Epac1camps*/+;UAS*ACXD*/+	69	28	24.7±0.08	41.3±3.4
*Pdf*-gal4/y;*Epac1camps*/+;UAS*rut*/+	22	27	23.8±0.04	47.5±6.4
*KK: Gsa60ARNAi/+*	23	4	24.1±0.20	45.2±4.7
*UAS-Gsα60A/+*	34	0	24.3±0.5	110.5±5.5
*Pdf*-gal4/y;*Epac1camps*/KK:*Gsα60A*RNAi	24	0	25.0±0.06	60.2±6.2
*Pdf*-gal4/y;*Epac1camps*/UAS*Gsα60A*	56	64	24.9±0.27	22.3±3.1
TRiP:*nervy*RNAi/+	26	15	23.4±0.17	61.1±7.7
*Pdf*-gal4/y;*Epac1camps*/+;TRiP:*nervy*RNAi/+	34	29	23.8±0.09	48.2±4.9
*Pdf*-gal4/y;*Epac1camps*/KK:*rugose*RNAi;+/+	22	32	25.2±0.16	60.0±5.8
*Pdf*-gal4/y;*Epac1camps*/+;TRiP:*AKAP200*RNAi/+	20	20	24.5+0.03	60.0±0.9

Periods are calculated using chi-squared periodigram. Flies with a power <10 were scored as arrhythmic.

## Discussion

Networks of pacemaker cells are synchronized by intercellular interactions [3],[9],[19]. There is strong and diverse evidence that control of cAMP levels is a critical factor underlying pacemaker rhythmicity and synchronization. Daily changes in cAMP levels in SCN neurons contribute to setting the phase, period, and amplitude of PER2 cycles and thus represent an integral component of the clock mechanism itself [37]. Furthermore, the RGS16 regulator sets the level of cAMP generation and its levels are likewise clock-controlled [38]. Regarding synchronizing agents that couple diverse pacemakers, both PDF in the fly and VIP in the mouse produce cAMP increases in response to receptor activation, and these signals ultimately have access to the pacemaker mechanism in target cells [4],[9],[10],[39]–[41]. Thus understanding the molecular components that control cAMP metabolism in pacemaker neurons, especially those downstream of receptors for the PDF and VIP modulators, are significant goals for the field.

There are at least 12 different genes encoding adenylate cyclases in the fly genome, of which the best known is Rutabaga, a calcium- and calmodulin-sensitive AC. Rut was first identified in a screen for mutations that affected learning and memory exhibited in an associative conditioning paradigm [29]. The Rut cyclase displays the properties of a coincidence detector with its activity triggered by inputs from simultaneous activation of more than one GPCR [24]. However, our studies indicate that, in M pacemakers, the PDF receptor is preferentially coupled not to Rut but to the adenylate cyclase encoded by *AC3*. In vitro studies suggest the AC3 cyclase may be inhibited by calcium [42]. The functional consequences of this specific signaling association, the physical basis that supports it, and the degree to which it may hold true in other PDF-responsive neurons in the *Drosophila* brain are important questions raised by this work.

The experiments that manipulated AC and PDF-R expression together indicate that relative levels of AC enzyme and receptor are important determinants of normal PDF cAMP responses in M pacemakers. Counterintuitively, *AC3* over-expression was as effective in diminishing PDF responsiveness as was *AC3* knockdown. One possible explanation is that the abnormally high levels of AC3 result in incorrect subcellular localization of signaling components, which may preclude the ability of AC3 to contribute to cAMP generation. Within M cells, only moderate expression of a UAS-*AC3* transgene could restore normal PDF responses after knockdown of endogenous *AC3*. Likewise, over-expressing AC3 together with PDF-R could restore the balance between receptor and effector, as indicated by the return of PDF responsiveness. Although these results may not generalize to all cell types or receptor pathways, it is notable that, for this circadian signaling pathway, appropriate levels of signaling components were as important as their simple presence or absence. The reliance on proper stoichiometry between receptor and AC is further evidence to support the hypothesis that PDF-R and AC3 exhibit a specific functional association within the M class of pacemaker cells.

**Figure 9:**
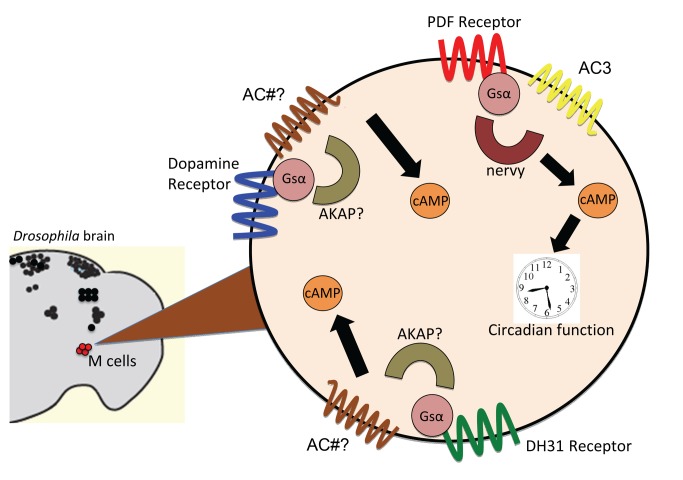
A model for a circadian signalosome comprised of preferential PDF-R:AC3:nervy coupling. M pacemakers respond to dopamine, DH31, and PDF-R through G_s_α-coupled receptors: Activation of each receptor lead to increases in cAMP levels. Both DH31 and PDF receptors signal through *G_s_α60A*, however AC3 alterations affect PDF signaling without affecting DH31 responses. We propose that PDF signals through AC3 to affect circadian function but that dopamine and DH31 couple to other AC isoforms. Likewise, the AKAP nervy preferentially associates with the PDF-R:AC3 signaling complex; other AKAPs support the G_s_α-coupled receptors that mediate responsiveness to DA and DH31.

One possible explanation for preferential coupling of PDF-R to AC3 is simply that it is the only adenylate cyclases to be expressed in M cells. However this explanation is inconsistent with at least two notable observations—first, M cells in flies with a severe *AC3* knockdown (*Df2L;GD:AC3RNAi*) still elevate cAMP levels normally in response to neuropeptide DH31. Second, according to recent profiling studies, multiple other ACs are normally expressed at appreciable levels in larval LNs [43] and in adult LNv [34]. Interestingly, these studies indicate that *AC3* is not even the most abundant adenylate cyclase [34]. Therefore, we favor an alternative explanation—that molecular specificity dictates the composition of different receptor pathways, with PDF-R residing in privileged association with AC3 (Figure 9).

There is clear support for the concept of preferential coupling between GPCRs and specific ACs in multiple cell types, in addition to our own findings in *Drosophila* clock cells. Previous work in *Drosophila* [44] suggests that individual cyclases play specific roles in G-protein signaling associated with gustation. Furthermore, studies of the GABAergic system in the mouse pituitary indicate that Type 7 adenylate cyclase is associated with ethanol and CRF sensitivity, although mRNA for four of the nine mammalian ACs are detected by microarray in pituitary tissue [45],[46]. It has also been proposed that receptor/AC preference may depend upon environmental conditions: for example, the Type 7 preference of the CRF receptor in the mouse amygdala occurs only after phosphorylation of signaling components. Without phosphorylation, CRF receptor couples preferentially to Type 9 adenylate cyclase [47]. Thus, our results add to the body of evidence that highly specific associations between receptors and their downstream partners are key regulators of signaling.

There is clear evidence that signaling components within specific pathways do cluster, which may explain how generalized signaling molecules like cAMP and PKA are capable of targeting distinct downstream effectors. Much current work focuses on possible mechanisms for such localization, [35] and the concept of signalosomes has been proposed to describe the spatial sequestering of signaling pathway components to promote exactly this sort of specific association. Thus preferential AC3/PDF-R coupling may be achieved by localizing AC3 near to PDF receptors. Mechanisms for grouping signaling components may include their co-localization in lipid rafts; many of the components of cAMP signaling including G proteins, PDE, PKA, and cyclic nucleotide gated channels are found in lipid rafts [48] and studies in human bronchial smooth muscle cells detected three different AC isoforms, which are present in distinct membrane microdomains and which respond to different neurotransmitters and hormones [49].

In addition, it is likely that another clustering mechanism includes the formation of macromolecular structures through the use of scaffolding proteins that bind to signaling molecules, as first proposed by Stadel and Crooke [50]. Later studies showed that ACs form large complexes with *β*-arrestins, G proteins, and calcium channels [51]. The scaffolding protein InaD is required for normal localization of signaling components in the fly visual system including TRP and PLC [52]–[54]. Specialized signaling components such as AKAPs (A-kinase anchoring proteins) can bind to receptors as well as kinases and adenylate cyclases [35]. In *Drosophila*, AKAPs organize functionally discrete pools of PKA, and disruption of these signaling complexes alters normal spatio-temporal signal integration and causes a loss of anesthesia-sensitive as well as long-term olfactory memory formation in flies [55],[56]. In our study, knockdown of AKAP *nervy* reduced PDF responses: These results lead to a hypothesis whereby, in M pacemakers, PDF receptor preferentially couples to AC3 via a nervy-based scaffold system to produce normal circadian behavior (Figure 9). We emphasize that, while our results demonstrate a functional connection between AC3 and PDF-R, the basis for any physical connections has not yet been established.

Although our study provides an example of a specific receptor/enzyme pairing in a subset of circadian clock cells, our evidence also suggests the exact details of PDF signaling in other *Drosophila* pacemakers may differ. Simply put, the set of AC3 manipulations that caused a disruption of PDF responsiveness in M pacemakers had no such effect in E pacemakers. Importantly, disruption of Gsα affected both subgroups (see Figures 5A and 7B). Multiple lines of evidence have suggested that PDF signaling differs between clock cell subgroups. (i) Loss of PDF has distinct effects on PERIOD protein cycling in LNv (M cells) versus non-LNv cells (E cells). Both cell groups continued to show cycling in PER immunostaining levels and localization but, while M cells become phase-dispersed in PER cycles, E cells remain synchronized with altered phase and amplitude of PER accumulation [9],[16]. (ii) In *Pdf/cry* and *PDF-R/cry* double mutants, a subset of E cells show a severe attenuation of the PER molecular rhythm, while M cells continue to cycle normally [41],[57],[58]. Different subsets of E cells have previously been implicated in control of evening anticipation, and even when AC3 is altered in all clock cells, the evening peak retains its proper phase, again suggesting that AC3 is not a required enzyme in E type pacemaker cells (unpublished). These finding are consistent with the hypothesis that there are two functionally different PDF signaling pathways. However, although we have confirmed that adenylate cyclases are responsible for the PDF FRET responses in E cells (Figure S2), as yet we have no positive evidence regarding the contribution of any single AC in E pacemakers (unpublished data). Hence it remains to be determined how uniform the components of PDF signalosomes in the M versus E pacemaker cell types are.

How well do the observations obtained with neuronal imaging predict or correlate with circadian locomotor behavior? Manipulations of *AC3* that severely disrupt PDF signaling in M cells were correlated with a loss of morning anticipation and increased arrythmicity in DD. Manipulations that only partially reduce the FRET response (for example, single *AC3* or single *nervy* knockdown) resulted in normal circadian locomotor behavior or disruptions to some aspects but not all. The latter observations suggest that the animal is capable of compensating for reduced AC3-generated cAMP responses by M cells but not to complete loss of AC3 function (see Tables 1 and 2 and Figure S5). These data argue for a contribution to behavior by PDF signaling via AC3 in M cells and stand in contrast to a recent report by Lear et al. [10]. That group reported that PDF-R expression in E cells alone is sufficient for morning anticipation and that exclusive expression of PDF-R in M cells does not recover morning anticipation. We cannot reconcile these differences without further experimental efforts, but note that GAL80 techniques are not always sufficient to extinguish gene expression in vivo (unpublished data).

Depending on ambient conditions [12],[57], the M cells contribute to normal morning anticipatory behavior and to maintenance of rhythmicity under constant dark conditions [2],[14],[20],[58]–[60]. However, in our study M cells expressing *AC* RNAi remain normally responsive to at least two other neurotransmitters (DH31 and dopamine). Hence we suspect that much of the behavioral effect of knocking down *AC3* in M pacemakers is mainly due to loss of PDF signaling in them despite retention of additional inputs from a PDF-independent source. Levels of PDF receptor and responsiveness to PDF are both high in small LNv cells and absent (or barely detectable) in large LNv cells [19],[20],[34]. Therefore we expect that *AC3* alterations in M cells (directed by *Pdf*-GAL4) primarily affect PDF signaling in LNvs. In these considerations, the extent to which the AC3 behavioral phenotype is explained by PDF-R coupling to AC3 in M cells is not yet defined. AC3 appears coupled to at least one other GPCR pathway in LNvs because, in DD, *AC3* knockdowns produced a more severe behavioral phenotype than did *Pdf* null flies (a higher percentage of arrhythmicity).

Knockdown of Gsα60A levels of the M pacemakers lengthened the period in DD, a behavioral effect opposite to those seen following loss of PDF, or M cell ablation, namely. Previous studies of Gsα60A in M cells also reported a long period phenotype [43]. Likewise selective expression in small LNv of *shibiri* (a dominant negative allele of the fly homolog to dynamin [61]) or of a chronically open sodium channel [60] both produce long period phenotypes [62],[63]. Although we cannot rule out a PDF-dependent role in period lengthening in our Gsα60A experiments, our imaging data suggest the lengthened period phenotype may be explained by the fact that alterations of Gsα60A impact multiple signaling pathways (see Figure 5).

Our results demonstrate in *Drosophila* that, in small LNv (M) circadian pacemakers, a highly specific signaling cascade is activated in response to PDF. They suggest there exists a dedicated PDF-R::AC3-dependent signaling pathway that functions to synchronize these particular clock cells. A different PDF signaling cascade is likely to operate in E pacemakers. The complete molecular details of these signaling complexes, their convergence with CRY signaling [41], and their ultimate connections to the cycling mechanism are significant issues for future studies.

## Materials and Methods

### Fly Rearing and Stocks

*Drosophila* were reared on cornmeal/agar supplemented with yeast and reared at 25°C, unless otherwise indicated by experimental design. Male flies (age 2 to 5 d old) were moved to 29°C for 24–48 h before imaging to increase UAS transgene expression. For temperature shift (tubulin-gal80*ts*) experiments, crosses were maintained at 18°C to maintain gal80*ts* suppression of gal4, and males were collected and moved to 29°C for 24–48 h before imaging to allow UAS transgene expression. For temperature shift UAS*AC3*/TRiP:*AC3*RNAi rescue experiments, males were reared at 25°C and moved to 18°C for 12–16 h before imaging to reduce gal4-driven expression of AC3. All gal4 lines used in this study have been described previously: *Pdf*(m)-gal4 [64], UAS- *Epac1camps*50A [20], and *Mai179*-gal4 [65]. The TRiP:RNAi (UAS-TRiP:*AC3*RNAi, UAS-TRiP:-*nervy*RNAi, UAS-TRiP:*AKAP200*RNAi), UAS *Gsα60A*, UAS-*rutabaga*, tubulin-gal80*ts*, and Df(2)*LDS6* lines were obtained through the Bloomington Stock Center (thanks to the Harvard TRiP RNAi project) and the UAS-*Gsα60A*RNAi, UAS-GD:*AC3*RNAi, UAS-*AC13*ERNAi, UAS- *AC78C*, UAS-*rut*RNAi, UAS-*ACXA*RNAi, UAS*ACXB*RNAi, UAS-*ACXC*RNAi, and UAS*ACXD*RNAi. UAS-*yu*RNAi and UAS-*rugose*RNAi lines were obtained through the Vienna RNAi Stock Center.

### Live Imaging

For epifluorescent FRET imaging, living brains expressing gal4-driven uas-*Epac1camps* were dissected under ice-cold calcium-free fly saline (46 mM NaCl, 5 mM KCl, and 10 mM Tris (pH 7.2)). All lines tested included one copy each of gal4 (*Pdf*-gal4 used for small LNv cells and Mai179gal4 for PDF-R(+)LNd cells) and *Epac1camps*. All genotypes include one copy of each transgene unless otherwise indicated. Full genotypes are available in Table S1. For the RNAi AC screen and for pharmacological experiments, whole brains were placed at the bottom of a 35×10 mm plastic FALCON Petri dish (Becton Dickenson Labware) as in [20], incubated in HL3 saline, and substances tested by bath application. For all remaining experiments, dissected brains were placed on poly-l-lysine-coated coverslips in an imaging chamber (Warner Instruments), and HL3 was perfused over the preparation (0.5 mL/minute). Microscopy was performed through a LUMPL 60×/1.10 water objective with immersion cone and correction collar (Olympus) on a Zeiss Axioplan microscope. Excitation and emission filter wheels were driven by a Lambda 10-3 optical filter changer and shutter control system (Sutter Instrument Company) and controlled with SLIDEBOOK 4.1 software (Intelligent Imaging Innovations). Images were captured on a Hamamatsu Orca ER cooled CCD camera (Hamamatsu Photonics). Exposure times were 20 ms for YFP- FRET and 500 ms for CFP donor. Live FRET imaging was performed on individual cell bodies, YFP-FRET and CFP donor images were captured every 5 s with YFP, and CFP images were captured sequentially at each time point. Following 45 s of baseline YFP/CFP measurement the peptide was bath added/injected into the perfusion line to result in a final concentration of 10^−06^ M. FRET readings were then continued to result in a total imaging time course of 10 min. ODQ and dopamine were purchased from Sigma. Synthetic DH31was provided by David Schooley and PDF was produced by (Neo MPS, San Diego, CA, USA).

### Imaging Data Analysis

For all experiments reported, we collected responses from at least 10 cells that were found in at least five brains for all genotypes. A region of interest (ROI) defined each individual neuron, and for each, we recorded background-subtracted CFP and YFP intensities. The ratio of YFP/CFP emission was determined after subtracting CFP spillover into the YFP channel from the YFP intensity as in [26]. The CFP spillover (SO) into the YFP channel was measured as .397 [20]. For each time point, FRET was calculated as (YFP−(CFP * SO CFP))/CFP. To compare FRET time courses across different experiments, FRET levels were normalized to initial baseline levels and smoothed using a 7-point boxcar moving average over the 10-min imaging time course. Statistical analysis was performed at maximal deflection from the initial time point by performing ANOVA analysis followed by post hoc Tukey tests using Prism 5.0 (Graphpad Software Inc.).

### Over-Expression Constructs

Over-expression constructs were built by PCR construction from cDNA derived from adult heads (*Canton S*) and subcloned into *P{cDNA3}* and *P*{UAS-*attb*} vectors. The original *AC3* clone was a kind gift from Lonny Levin (Weill Cornell Medical College).

The sequences of all primers used in this study are: AC3(BamHI) 5′: GGATCCATGGAAGCAAATTTGGAGAACGGTC; AC3(EcoRV) 3′: GATATCCTATTCTAGCAAAGACTGACATTCT; AC78C 3′: CTATAACGCATCGTTGTGGCTCTTCGATAT; AC78C nested 3′: ACTTAGACCCAGTGAGTGCGCGTACTCGG ; AC78C 5′: ATGGACGTGGAACTCGAAGAGGAGGAGGAG; AC78C nested 5′: GCATAGCAATAGACAGAATCCTCCGCCACA; AC76E 3′: CTACAATTTCCCATCGAAAGGTGTCTTTAC; AC76E nested 3′: ATCAACAGCAACTGGGTGACGATCGGTGAT; AC76E 5′: ATGGTAAATCACAATGCGGAAACTGCGAAA; AC76E nested 5′: GCCACTAGCTACACGCCACCGCTTTTCGCC; ACXD5′: ATGGACTCCTACTTCGACTCGGCC; and ACXD3′: CTAGTCTTCTTTGGTTGGCGCGGCC.

### In Vitro Signaling Assays

*hEK-293* cells were tested using a *cre*-LUC reporter in response to 10 µM forskolin 24 h post-transfection with different UAS-*AC* constructs that had been subcloned into p{*CDNA3*}. All constructs were co-transfected with *cre-luc* and compared to empty- vector-transfected cells (0.5 µg *cre-luc* and 2.5 µg PDF-R and 2.5 µg AC). Four hours after forskolin addition, cells were lysed and luciferin added, followed by bioluminescence measurement using a Victor-Wallac plate reader. Measurements were performed in triplicate and normalized to vehicle-treated controls; the results represent combined activities from three independent transfections.

### Locomotor Activity

Male flies were loaded into Trikinetics Activity Monitors 4–6 d after eclosion. Locomotor activities were monitored for 6 d under 12∶12 light/dark and then for 9 d under constant darkness (DD) conditions. Anticipation index was calculated as in [19] as (activity for 3 h before lights-on)/(activity for 6 h before lights-on). To analyze rhythmicity under constant conditions we normalized activity from DD Days 3–9 and used *X_2_* periodogram with a 95% confidence cutoff as well as SNR analysis [66]. Arrhythmic flies were defined by having a power value <10.

## Supporting Information

Figure S1 PDF signals through cAMP, not cGMP, in M cells. (A) Pretreatment of brains with guanylate cyclase inhibitor (ODQ) has no effect on PDF responses. (B) Pretreatment of brains with guanylate cyclase inhibitor (ODQ) significantly reduces SNAP responses. (C) Over-expression of cAMP-specific phosphodiesterase dunce significantly reduces PDF response. (D) Over-expression of cAMP-specific phosphodiesterase dunce has no effect on SNAP responses. All genotypes include *Pdf*-gal4;*Epac1camps*. Error bars denote SEM. *** *p*<0.001 (compared with control).
(TIF)

Figure S2 PDF signals through cAMP, not cGMP, in E cells. (A) Pretreatment of brains with guanylate cyclase inhibitor (ODQ) has no effect on PDF responses. (B) Pretreatment of brains with guanylate cyclase inhibitor (ODQ) significantly reduces SNAP responses. (C) Over-expression of cAMP-specific phosphodiesterase dunce significantly reduces PDF response. (D) Over-expression of cAMP-specific phosphodiesterase dunce has no effect on SNAP responses. All genotypes include *Mai179*-gal4;*Epac1camps*. Error bars denote SEM. *** *p*<0.001 (compared with control).
(TIF)

Figure S3 AC over-expression in hEK cells. *Cre*-luc responses to forskolin in *hEK*-293 cells after transfection with AC over-expression constructs normalized to vehicle-treated cells.
(TIF)

Figure S4 Adult-only manipulations of AC3 and nervy reduce PDF responses in M cells. Both UAS*AC3* and TRiP:*nervy*RNAi reduce PDF responses in small LNv cells when expressed only in adult cells. All genotypes include *Pdf*-gal4;*Epac1camps*. Error bars denote SEM. *** *p*<0.001 (compared with control).
(TIF)

Figure S5 LD Actograms of genotypes with partial reduction of M cell FRET response. (A) Representative locomotor behavior of flies expressing a single copy of GD:*AC3*RNAi. (B) Representative locomotor behavior of flies expressing a single copy of TRiP:*AC3*RNAi. (C) Representative locomotor behavior of flies expressing a single copy of GD:*AC76E*RNAi. (D) Representative locomotor behavior of flies expressing a single copy of TRiP:*nervy*RNAi. Average morning anticipation index was calculated from three replicates for each genotype. Error bars denote SEM. * *p*<0.05, ** *p*<0.01 (compared with control). Statistical analysis for morning anticipation is shown in Table 1, and behavioral outcomes for DD are shown in Table 2.
(TIF)

Table S1 Full genotypes for all lines tested, listed by figure.
(XLSX)

## Abbreviations

AC: adenylate cyclase
AKAP: A-kinase anchoring protein
cAMP: cyclic adenosine monophosphate
CFP: cyan fluorescent protein
cGMP: cyclic guanosine monophosphate
DA: dopamine
DH31: Diuretic Hormone 31
DH31-R: Diuretic Hormone 31 Receptor
EPAC: Exchange Protein Activated by cAMP
FRET: Förster Resonance Energy Transfer
GPCR: G-protein coupled receptor
LNd: dorso-lateral neuron
LNv: ventro-lateral neuron
NO: Nitric Oxide
ODQ: 1H-[1,2,4]Oxadiazolo [4,3-a]quinoxalin-1-one
PDF: Pigment Dispersing Factor
PDF-R: Pigment Dispersing Factor Receptor
rut: rutabaga
SNAP: S-Nitroso-N-acetyl-DL-penicillamine
VDRC: Vienna *Drosophila* RNAi Center
YFP: yellow fluorescent protein
