# Supplementary figures and images for: The Circadian Neuropeptide PDF Signals Preferentially through a Specific Adenylate Cyclase Isoform AC3 in M Pacemakers of Drosophila
Source: PLoS Biol. 2012 Jun 5;10(6):e1001337. doi: 10.1371/journal.pbio.1001337 (PMC3367976; doi:10.1371/journal.pbio.1001337)

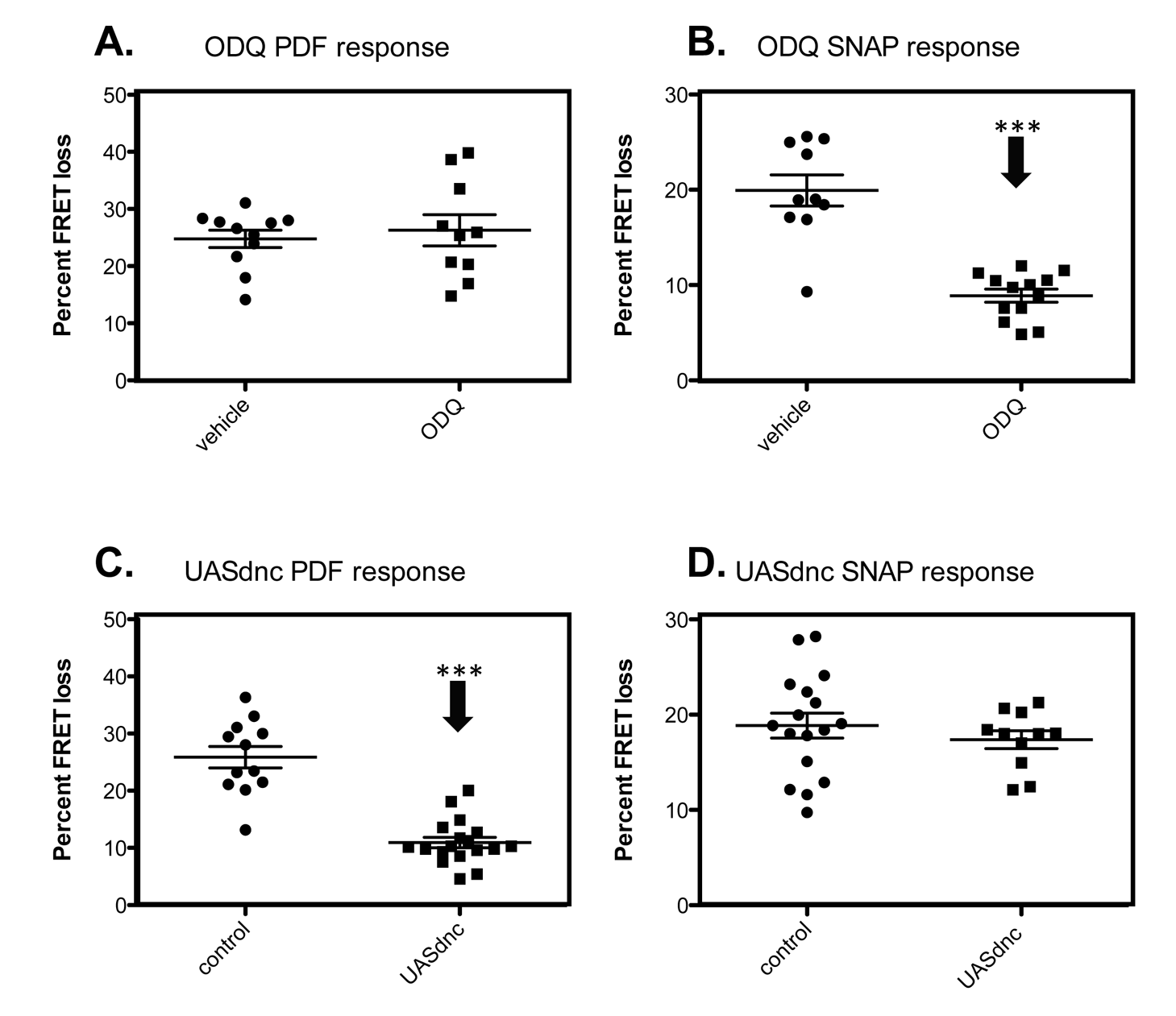

Supplement: Figure S1 — PDF signals through cAMP, not cGMP, in M cells. (A) Pretreatment of brains with guanylate cyclase inhibitor (ODQ) has no effect on PDF responses. (B) Pretreatment of brains with guanylate cyclase inhibitor (ODQ) significantly reduces SNAP responses. (C) Over-expression of cAMP-specific phosphodiesterase dunce significantly reduces PDF response. (D) Over-expression of cAMP-specific phosphodiesterase dunce has no effect on SNAP responses. All genotypes include Pdf-gal4;Epac1camps. Error bars denote SEM. *** p<0.001 (compared with control). (TIF) [file pbio.1001337.s001.tif]

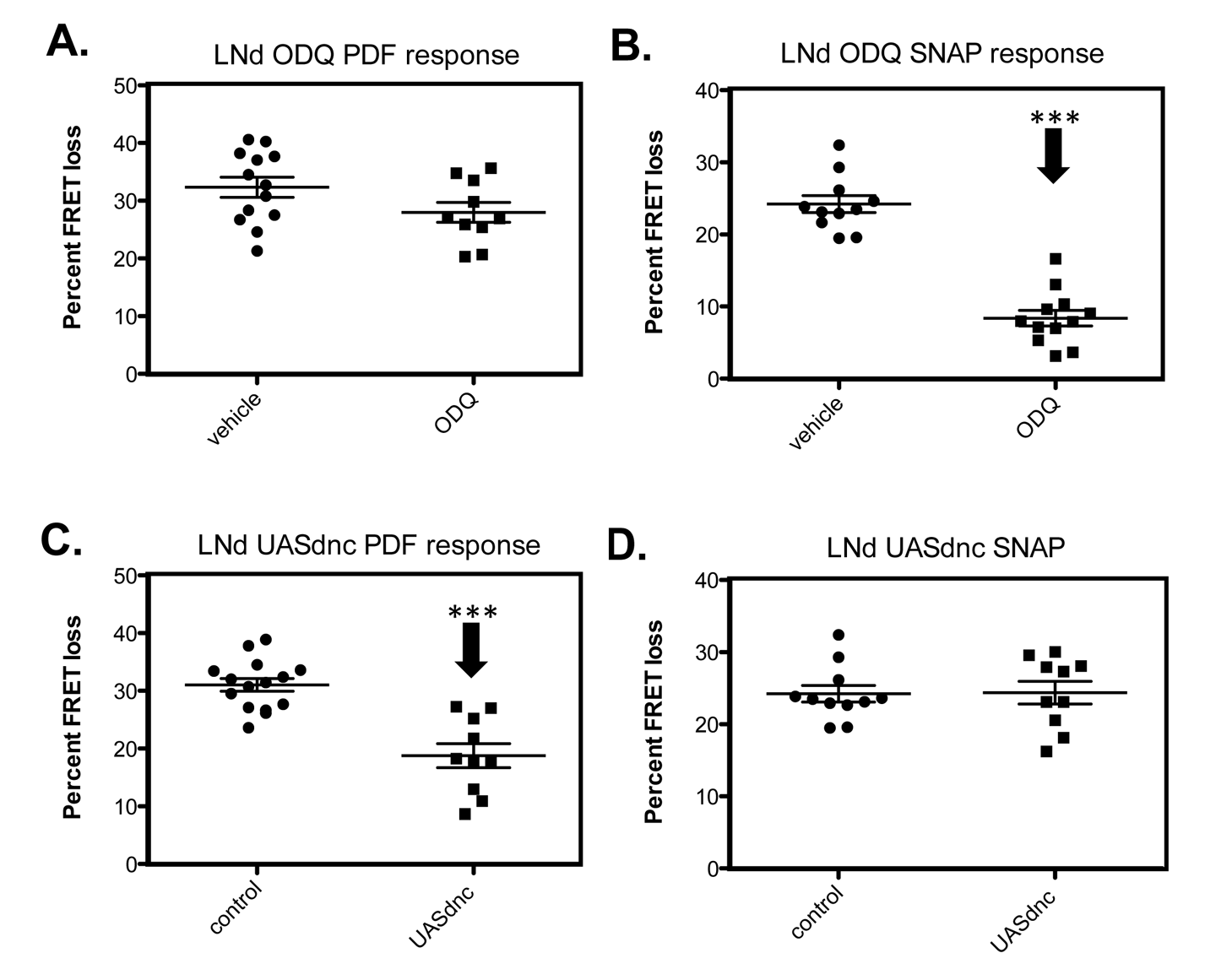

Supplement: Figure S2 — PDF signals through cAMP, not cGMP, in E cells. (A) Pretreatment of brains with guanylate cyclase inhibitor (ODQ) has no effect on PDF responses. (B) Pretreatment of brains with guanylate cyclase inhibitor (ODQ) significantly reduces SNAP responses. (C) Over-expression of cAMP-specific phosphodiesterase dunce significantly reduces PDF response. (D) Over-expression of cAMP-specific phosphodiesterase dunce has no effect on SNAP responses. All genotypes include Mai179-gal4;Epac1camps. Error bars denote SEM. *** p<0.001 (compared with control). (TIF) [file pbio.1001337.s002.tif]

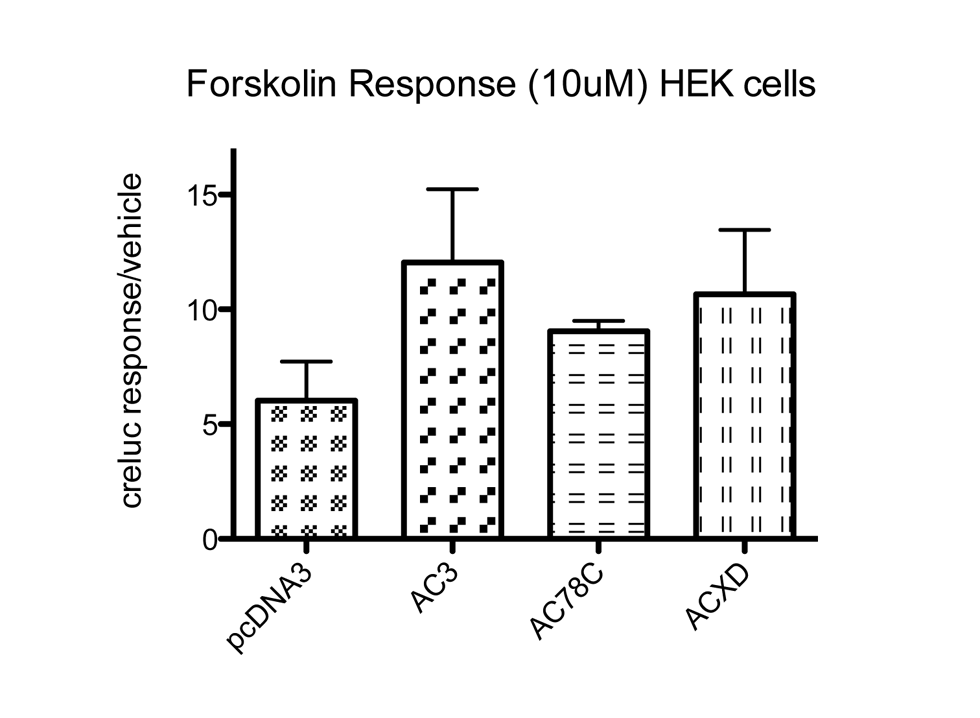

Supplement: Figure S3 — AC over-expression in hEK cells. Cre-luc responses to forskolin in hEK-293 cells after transfection with AC over-expression constructs normalized to vehicle-treated cells. (TIF) [file pbio.1001337.s003.tif]

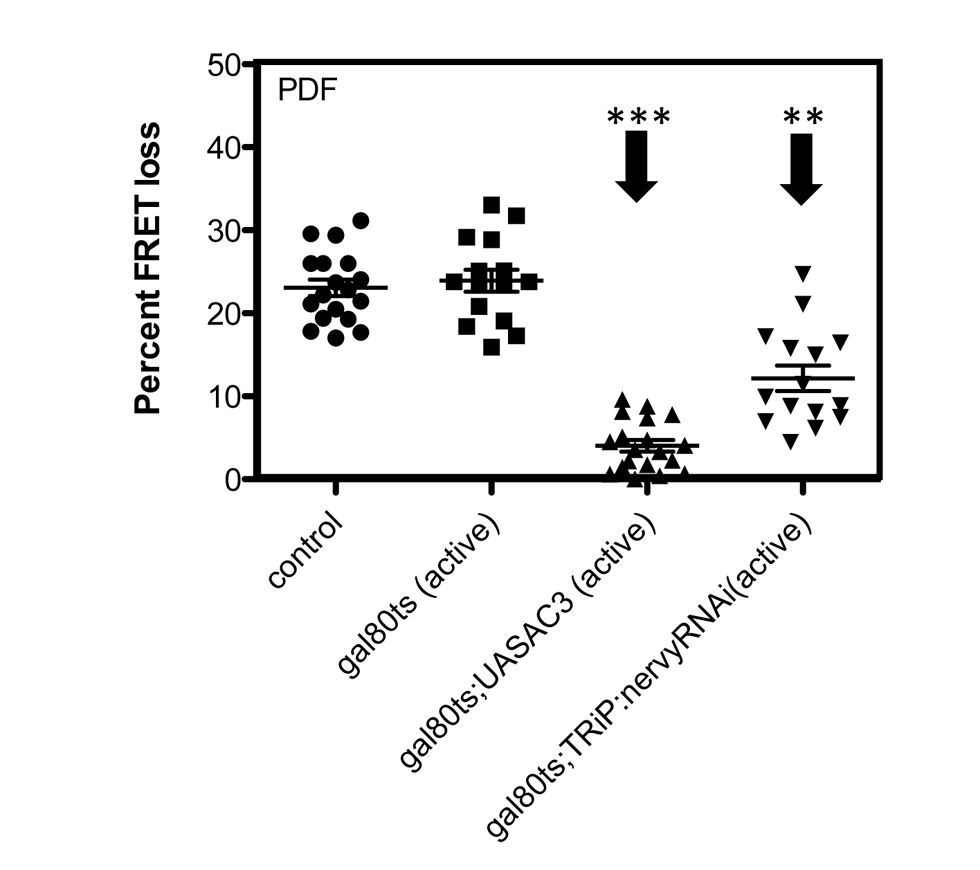

Supplement: Figure S4 — Adult-only manipulations of AC3 and nervy reduce PDF responses in M cells. Both UASAC3 and TRiP:nervyRNAi reduce PDF responses in small LNv cells when expressed only in adult cells. All genotypes include Pdf-gal4;Epac1camps. Error bars denote SEM. *** p<0.001 (compared with control). (TIF) [file pbio.1001337.s004.tif]

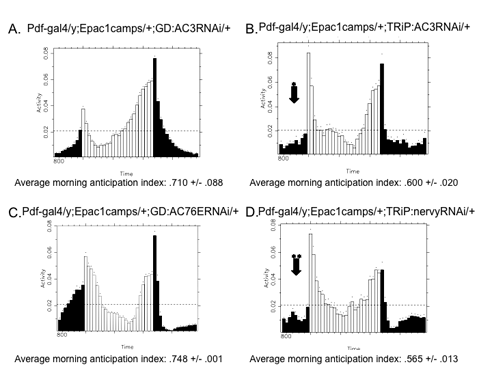

Supplement: Figure S5 — LD Actograms of genotypes with partial reduction of M cell FRET response. (A) Representative locomotor behavior of flies expressing a single copy of GD:AC3RNAi. (B) Representative locomotor behavior of flies expressing a single copy of TRiP:AC3RNAi. (C) Representative locomotor behavior of flies expressing a single copy of GD:AC76ERNAi. (D) Representative locomotor behavior of flies expressing a single copy of TRiP:nervyRNAi. Average morning anticipation index was calculated from three replicates for each genotype. Error bars denote SEM. * p<0.05, ** p<0.01 (compared with control). Statistical analysis for morning anticipation is shown in Table 1, and behavioral outcomes for DD are shown in Table 2. (TIF) [file pbio.1001337.s005.tif]
